# Lack of *tailless* leads to an increase in expression variability in *Drosophila* embryos^☆^

**DOI:** 10.1016/j.ydbio.2013.01.010

**Published:** 2013-05-01

**Authors:** Hilde Janssens, Anton Crombach, Karl Richard Wotton, Damjan Cicin-Sain, Svetlana Surkova, Chea Lu Lim, Maria Samsonova, Michael Akam, Johannes Jaeger

**Affiliations:** a EMBL/CRG Research Unit in Systems Biology, CRG—Centre de Regulació Genòmica, and Universitat Pompeu Fabra (UPF), Dr. Aiguader 88, 08003 Barcelona, Spain; b Department of Computational Biology, Center for Advanced Studies, St. Petersburg State Polytechnical University, 29 Polytehnicheskaya Street, St. Petersburg 195251, Russia; c Department of Zoology, Downing Street, Cambridge CB2 3EJ, UK

**Keywords:** *Drosophila* embryogenesis, Segmentation gene network, Quantitative expression analysis, Pattern formation, Robustness/canalisation, Genetic capacitance

## Abstract

Developmental processes are robust, or canalised: dynamic patterns of gene expression across space and time are regulated reliably and precisely in the presence of genetic and environmental perturbations. It remains unclear whether canalisation relies on specific regulatory factors (such as heat-shock proteins), or whether it is based on more general redundancy and distributed robustness at the network level. The latter explanation implies that mutations in many regulatory factors should exhibit loss of canalisation. Here, we present a quantitative characterisation of segmentation gene expression patterns in mutants of the terminal gap gene *tailless* (*tll*) in *Drosophila melanogaster*. Our analysis provides new insights into the dynamic mechanisms underlying gap gene regulation, and reveals significantly increased variability of gene expression in the mutant compared to the wild-type background. We show that both position and timing of posterior segmentation gene expression domains vary strongly from embryo-to-embryo in *tll* mutants. This variability must be caused by a vulnerability in the regulatory system which is hidden or buffered in the wild-type, but becomes uncovered by the deletion of *tll*. Our analysis provides evidence that loss of canalisation in mutants could be more widespread than previously thought.

## Highlights

► We present a quantitative analysis of spatial gene expression in *Drosophila* mutants. ► Dynamic gap domain shifts do not depend on expression of terminal gap genes or *hb*. ► Expression variability is greatly increased in a *tll* mutant background. ► This indicates de-canalisation (loss of developmental robustness) in the mutant. ► Such de-canalisation is a common phenomenon in mutants of developmental regulators.

## Introduction

Developmental processes are generally robust. In other words, development exhibits canalisation: it produces constant phenotypic outcomes in the presence of genetic variation and changing environmental conditions (see Flatt, 2005, for review; Rendel, 1959; Waddington, 1942).

The cause of canalisation remains controversial. Studies of the Hsp90 chaperone suggest one explanation: if the dosage of Hsp90 is lowered, canalisation is lost, and many different mutant phenotypes can be observed in the affected population (Queitsch et al., 2002; Rutherford and Lindquist, 1998). It has been suggested that this effect is due to Hsp90’s position as a high-level hub in the regulatory network of the cell, and its function in tuning the activity level of several core signalling pathways (Rutherford et al., 2007). By lowering its dosage, these pathways can be pushed towards the sensitive range of their response thresholds, causing the observed discrete and stochastic distribution of different mutant phenotypes, which depends on the background genotypes present in the population. This indicates that specific factors can be responsible for a system’s robustness by suppressing existing genetic variability.

Computational studies, on the other hand, have suggested that developmental robustness need not be a property of single factors, but rather emerges at the level of gene regulatory networks (Bergman and Siegal, 2003; Siegal and Bergman, 2002; Wagner, 1996). Such networks exhibit redundant pathways – where one branch of a pathway can compensate for the loss of another – and/or distributed robustness – where mutations in one pathway can be compensated by other processes in the system (Wagner, 2005).

If canalisation were a distributed phenomenon, we would expect it to break down in mutants affecting factors other than Hsp90. Each mutation in a system – and in particular those affecting regulatory factors – can potentially reduce redundancy and distributed robustness, and render the system more vulnerable to further perturbations. Indeed, Bergman and Siegal (2003) observed widespread loss of canalisation, when they simulated large numbers of single (one-step) mutants of evolved, robust gene regulatory networks. Moreover, Levy and Siegal (2008) identified over 300 mutants in yeast, which exhibit reduced robustness to environmental fluctuations.

It is commonly known that many mutants affecting developmental processes in multi-cellular organisms exhibit increased levels of phenotypic variation compared to the wild-type. Variable penetrance of such mutations is a widely documented phenomenon (see Morgan, 1929, for an early example, and Burga et al., 2011, for a recent quantitative study). However, very little is known about the molecular mechanisms underlying the reduction or breakdown of canalisation in mutants of developmental regulators. The investigation of such mechanisms requires quantitative evidence at the level of gene expression and regulation. In this paper, we provide an example of such evidence: a quantitative characterisation of the *tailless* (*tll*) mutant in *Drosophila melanogaster*, which exhibits increased developmental variability compared to the wild-type. Similar results were obtained for mutants of *Krüppel (Kr)* and *knirps (kni)* in a parallel study by Surkova et al. (2013), which appears in this issue of Developmental Biology.

**Fig. 1:**
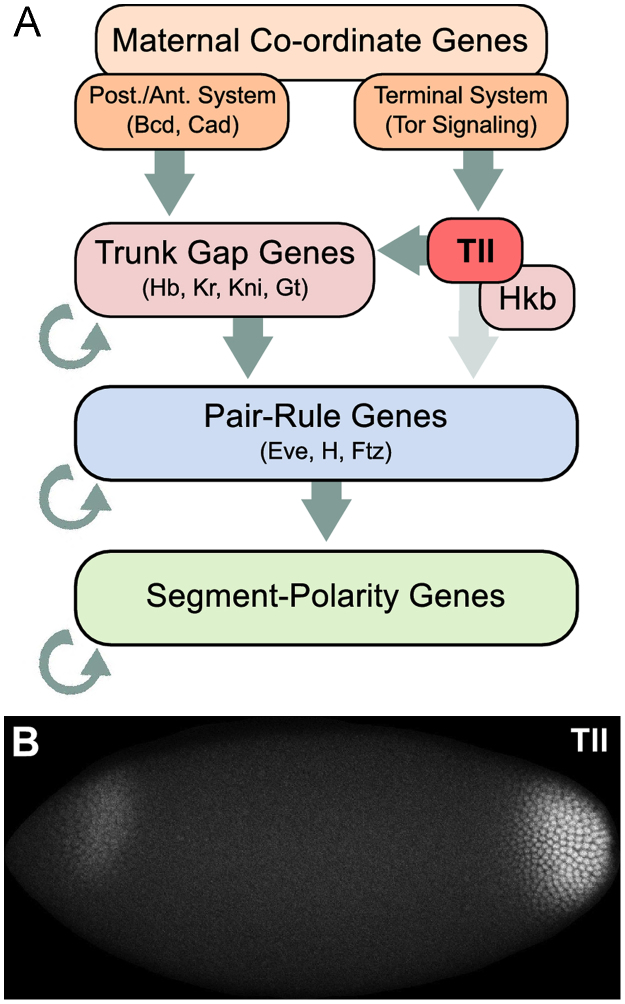
The role of Tll in the segmentation gene network in *Drosophila melanogaster*. (A) Schematic representation of the segmentation gene network. The terminal gap gene *tll* is regulated by the terminal maternal system, and (together with *hkb*) provides a feed-forward regulatory input on trunk gap and pair-rule genes. It is not part of any regulatory feedback loops. Circular arrows indicate cross-regulation among groups of genes. (B) Confocal image showing Tll protein expression in a wild-type embryo during the blastoderm stage (time class: t4). Lateral view. Anterior is to the left, dorsal is up. Image source: http://urchin.spbcas.ru/flyex.

The *tll* gene encodes a transcription factor of the nuclear hormone receptor family (Liaw and Lengyel, 1992; Pignoni et al., 1990). It is part of the segmentation gene network of *Drosophila*, which is involved in determining the positions of body segments during the blastoderm stage of early development (Fig. 1A; reviewed in Akam, 1987). More specifically, *tll* is a terminal gap gene: its mutants lack terminal structures, including the eighth abdominal segment, telson, and the posterior gut, as well as structures of the head and brain in the anterior (Jürgens et al., 1984; Pignoni et al., 1990, 1992; Strecker et al., 1986, 1988a,b). Its expression in the blastoderm is confined to two domains, one at the anterior and the other at the posterior pole of the embryo (Fig. 1B; Pignoni et al., 1990, 1992).

In concert with the other terminal gap gene *huckebein (hkb)*, *tll* conveys the regulatory input of the terminal maternal system to the trunk gap genes *hunchback (hb)*, *Krüppel (Kr), knirps (kni)* and *giant (gt)* (Fig. 1A; Klingler et al., 1988; Strecker et al., 1988a; Weigel et al., 1990). This regulation is entirely feed-forward: *tll* is not itself regulated by other gap genes (Brönner et al., 1994; Brönner and Jäckle, 1991). Instead it is activated, in a concentration-dependent manner, by the Torso (Tor) MAP-kinase signalling cascade in the anterior and posterior terminal regions (Brönner and Jäckle, 1991; Furriols et al., 1996; Greenwood and Struhl, 1997; Kim et al., 2010, 2011a,b; Pignoni et al., 1992). This activation is indirect, through repression of transcriptional repressors such as Capicua (Cic; Jiménez et al., 2000; Liaw et al., 1995). *tll*, in turn, represses the expression of *Kr*, *kni* and *gt*, while indirectly activating the posterior expression domain of *hb*, via repression of Kni, a potent repressor of *hb* (Casanova, 1990; Eldon and Pirrotta, 1991; Kraut and Levine, 1991; Morán and Jiménez, 2006; Pankratz et al., 1989; Reinitz and Levine, 1990; Steingrimsson et al., 1991). *tll* is also involved in regulation of downstream targets such as the pair-rule genes *even-skipped (eve)*, *hairy (h)*, and *fushi tarazu (ftz*; Fig. 1A), which all miss one of their seven expression stripes in *tll* mutants (Frasch and Levine, 1987; La Rosée et al., 1997; Mahoney and Lengyel, 1987; Riddihough and Ish-Horowicz, 1991).

The expression of gap, and other segmentation genes exhibits canalisation. It is robust towards both genetic and environmental perturbations. For example, most segmentation gene mutants do not show any sign of haploinsufficiency: except for minor cuticle defects in *Kr* heterozygotes (Wieschaus et al., 1984), severe dosage reduction causes no visible patterning defects (Nüsslein-Volhard and Wieschaus, 1980; see also the accompanying paper by Surkova et al., 2013). In the case of *hb*, the absence of both maternal and zygotic contributions can be rescued by one single paternal copy of the gene (Lehmann and Nüsslein-Volhard, 1987). Furthermore, expression of *hb* and *eve* are normal in embryos exposed to microfluidic temperature gradients that severely disrupt maternal regulators (Lucchetta et al., 2005, 2008). This suggests that the system is not only robust, but also self-correcting. Such self-correcting abilities are further supported by the fact that embryo-to-embryo variability in the position of domain boundaries decreases over time. While early gap gene mRNA expression patterns are extremely variable (Jaeger et al., 2007), they become precisely regulated to within one nucleus’ width only 1.5 h later, by the end of the blastoderm stage (Houchmandzadeh et al., 2002; Manu et al., 2009a, 2009b; Surkova et al., 2008a).

There are hints from the published literature, which suggest that mutations in the *tll* gene cause increased variability in expression features of its downstream regulatory targets. In particular, Frasch and Levine (1987) reported that a majority of *tll* mutants show only six stripes of Eve protein expression, but that a 7th stripe was observed in some embryos. However, this observation has never been investigated in a quantitative manner.

In this paper, we present our analysis of a quantitative data set of segmentation gene expression in a strain carrying the *tll*^*g*^ deficiency. Our data cover the expression dynamics of Bicoid (Bcd), Caudal (Cad), Hb, Kr, Kni, Gt, Hkb and Eve. We performed a careful comparison of our mutant expression data with wild-type segmentation gene patterns from the FlyEx database (http://urchin.spbcas.ru/flyex; Pisarev et al., 2009; Poustelnikova et al., 2004; Surkova et al., 2008a). This analysis provides new mechanistic insights into the dynamic regulation of gap domain boundaries, and detects significantly increased levels of expression variability at the late blastoderm stage in these embryos. This suggests a breakdown of canalisation in *tll* mutant embryos, probably caused by a cryptic sensitivity in the gap gene regulatory network, which becomes uncovered by the absence of the *tll* regulator.

## Materials and methods

### Fly stocks and husbandry

*tll* deletion strains *Df*(*3R*)*tll*^*g*^, *ca*^*1*^/*TM*3, *Sb*^*1*^ *Ser*^*1*^ (BS2599) and *Df*(*3R*)*tll*^*e*^, *ca*^*1*^*/TM6B*, *Tb*^*1*^ *ca*^*1*^ (BS5415), which were both identified in an X-ray screen (Strecker et al., 1988b), were ordered from the Bloomington stock center (http://fly.bio.indiana.edu). Wild-type Oregon-R and *eve* stripe 4+6(+1.5 kb to +6.6 kb) enhancer-*lacZ* flies (Sackerson et al., 1999) were a gift from John Reinitz. *eve* MSE3 (−3.8 kb to −3.3 kb) enhancer-*lacZ* flies (Small et al., 1996) were a gift from Stephen Small.

### Cuticle preparations

*Df*(*3R*)*tll*^*g*^, *ca*^*1*^/*TM3*, *Sb*^*1*^ *Ser*^*1*^ flies crossed to *w*⁎; *Sb*^1^/*TM*3, *P{ActGFP}JMR2*, *Ser*^*1*^ were allowed to lay eggs for 3 h in the dark at 25 °C. Embryos showing no detectable GFP signal at 18–21 h after egg laying (AEL) were collected, and their cuticles were prepared as described by Stern and Sucena (2011).

### Enzymatic *in situ* hybridisation

Wild-type and *tll*^*g*^ embryos were fixed 1–5 h after egg laying, and stained according to a colorimetric *in situ* hybridisation protocol adapted from Tautz and Pfeifle (1989) and Kosman et al. (2004). A detailed description of the protocol can be found in Crombach et al. (2012). *lacZ* riboprobe (Jiang et al., 1991) was labelled with DIG, *eve* riboprobe (Macdonald et al., 1986) with FITC.

### Quantitative gene expression data

All wild-type embryo images and expression profiles are from the FlyEx database (http://urchin.spbcas.ru/flyex; Pisarev et al., 2009; Poustelnikova et al., 2004) unless mentioned otherwise.

*tll* mutant embryos were stained for segmentation gene products and processed through a quantification pipeline involving the following steps: (1) antibody staining against Eve protein and 2 other segmentation gene proteins, (2) confocal scanning laser microscopy: each embryo was scanned using two optical sections separated by 1 μm, (3) image segmentation was performed to identify nuclei as described (Janssens et al., 2005; Surkova et al., 2008b, 2011), (4) time classification: embryos were assigned to cleavage cycle 13 (c13), or to one of the 8 time classes (t1–t8) of cleavage cycle 14A (c14A); each of the latter are a little over 6 min long (Myasnikova et al., 2001; see Fig. 2 of Surkova et al., 2008b), (5) removal of non-specific background staining (Myasnikova et al., 2005; Surkova et al., 2008b, 2011), (6) registration by spline approximation (Myasnikova et al., 2001; Surkova et al., 2008b, 2011) using the BREReA software (http://urchin.spbcas.ru/downloads/BREReA/BREReA.htm; successor of GCPReg, Kozlov et al., 2009), (7) extracting a strip along the lateral midline covering 10% of the embryo’s height, and (8) data integration: averaging of data for each gene and time-class, plus collection into 100 bins along the antero-posterior A–P axis; (Surkova et al., 2008b, 2011). (9) Integrated mutant and wild-type data were further smoothened by applying a Gaussian filter, and (10) mutant expression levels were scaled to the wild-type data to facilitate comparison between data sets, except for Hkb, where wild-type were scaled to *tll* mutant data.

Positions of gap gene protein domains in *tll* mutants, and of Eve protein stripes in *tll* mutant and wild-type embryos, were calculated as follows: position (along the A–P axis) of the maximum intensity of individual expression domains (domain peaks) was calculated by approximating the expression data with quadratic splines (Myasnikova et al., 2001). The positions of the anterior and posterior boundaries of each expression domain were calculated by extracting points of half-maximum fluorescence intensity from spline approximations. The width of a domain is defined by the distance (in % A–P position) between the position of the posterior boundary and the position of the anterior boundary of that domain as specified above. Position of gap domains in wild-type embryos were calculated in an equivalent way using dyadic wavelets instead of splines (Myasnikova et al., 2001).

*tll^-^/tll^-^* homozygous embryos were identified based on the Eve protein expression pattern in time classes t2–8. Younger *tll*^*-*^*/tll*^*-*^ embryos can only be distinguished from heterozygous or wild-type embryos through Tll antibody staining. Comparing early embryos with or without Tll staining shows that there are only very subtle differences in Eve expression in mutants versus wild-type before t2 (see Fig. 4). These differences could stem, at least in part, from difficulties to align embryos laterally at these early stages of development. Therefore, they were not analyzed further in this study.

### Statistical analysis of gene expression data

We used the statistical software package *R* (http://www.r-project.org) for assessing the equality of the mean (Student’s *t*-test) and variance (Levene’s test) of peak positions of eve stripes. In all cases, statistical tests were performed in two directions (“smaller than”, and “greater than”). Levene’s test was modified to use the median instead of the mean (also known as the Brown–Forsythe test) in order to accommodate for occasional small sample size and deviations from normality. Before statistical tests were performed, the (near) normality of the data was confirmed by visual inspection of quantile–quantile plots. After calculation, the *p*-values were corrected for multiple testing by using Bonferroni correction (*n*=168). Unless mentioned otherwise, corrected *p*-values <0.005 were regarded as statistically significant. Source code of *R* scripts is available upon request.

## Results

We acquired quantitative, spatial gene expression data from 1234 *tll*^*g*^ mutant embryos (587 of which were used in the final data set; see Table S1). Each embryo was stained for three proteins: Eve, and two other segmentation gene products. After confocal scanning, images were processed as described in the Materials and methods section. This resulted in a data set of 1773 expression profiles – at a nuclear spatial resolution and a temporal resolution of approximately 6–7 min – covering Bcd, Cad, Hb, Kr, Kni, Gt, Hkb, and Eve during cleavage cycles 13 and 14A (c13 and c14 A).

All results reported below were obtained with the *tll*^*g*^ deficiency strain. In addition, we also analyzed a smaller data set for the *tll*^*e*^ deficiency. Despite the fact that *tll*^*e*^ embryos are difficult to stage during late c14A – due to a cellularisation defect caused by the deletion of the *bottleneck (bnk)* gene (Schejter and Wieschaus, 1993) – results agreed between the two mutant strains (data not shown), indicating that the observed effects are unlikely to be caused by deleted genes other than *tll* itself, or second-site modifiers that depend on strain-specific genetic background.

### Analysis of Eve protein expression in *tll* mutant embryos

**Fig. 2:**
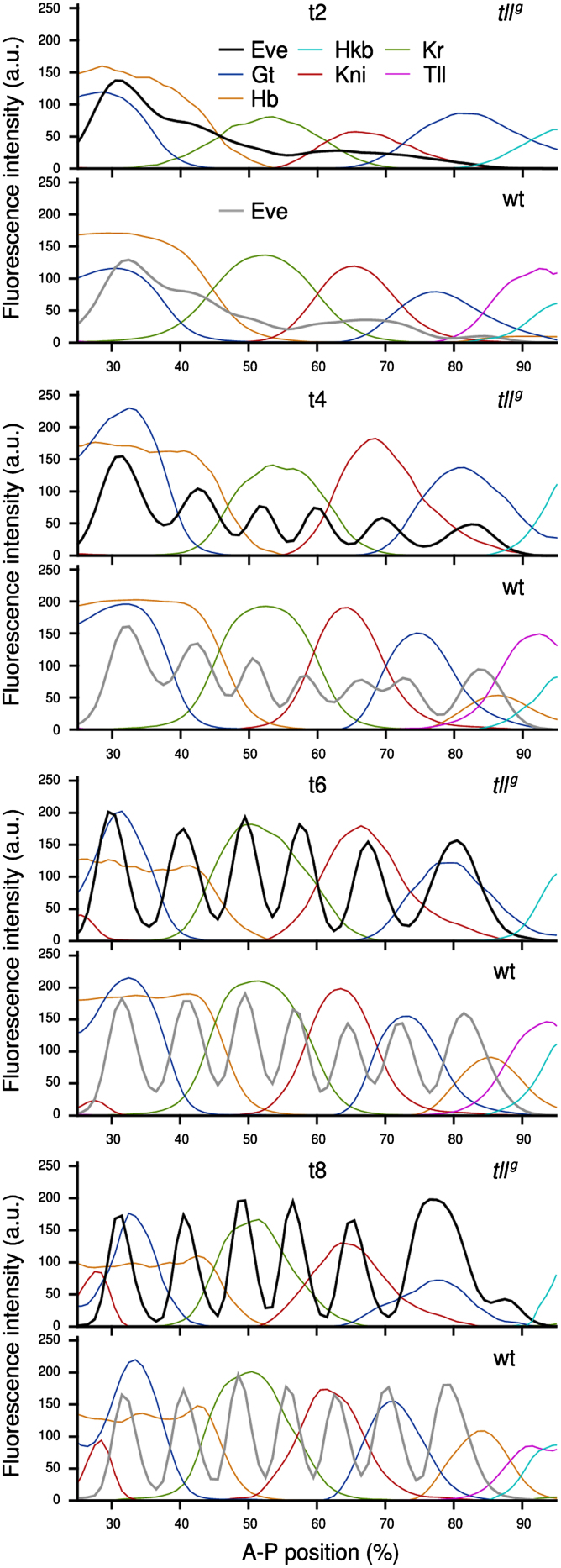
Relative positions of gap and Eve protein expression domains are maintained in wild-type versus *tll*^*g*^ embryos. Integrated (averaged) quantified expression patterns of Eve (grey/black), as well as gap gene products Gt, Hb, Hkb, Kni and Kr (coloured) are shown for wild-type and *tll*^*g*^ embryos at time classes t2, t4, t6 and t8. Vertical axes show fluorescence intensity in arbitrary units ranging from 0 to 255. Horizontal axes represent A–P position in percent (where 0% is the anterior pole). Only the trunk region of the embryo (35–92% A–P position) is shown. See the Materials and methods section for details on data quantification and time classification.

Frasch and Levine (1987) report that *tll*^*g*^ mutant embryos express Eve in 6 stripes, and only occasionally show a 7th stripe. Our quantitative gene expression data confirm this qualitative observation (Fig. 2; see also Figs. 4 and 6). Judging by previous evidence from enhancer-reporter assays (Fujioka et al., 1999; La Rosée et al., 1997) and the relative position of the Eve stripes with respect to the gap domains (Fig. 2; Surkova et al., 2008a), it is most likely stripe 7 that is missing.

**Fig. 3:**
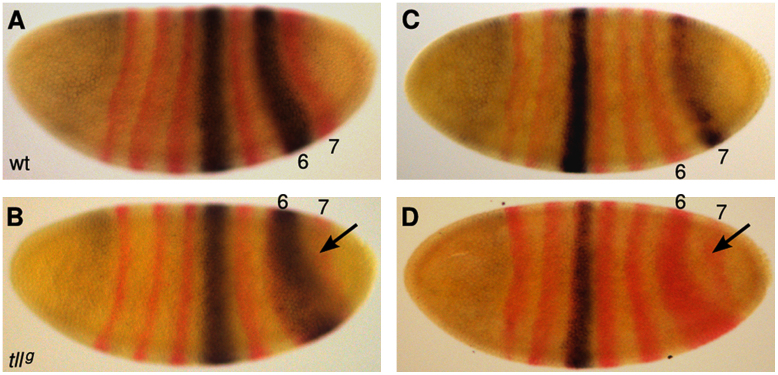
Expression driven by *eve* stripe enhancers in *tll*^*g*^ embryos. Wild-type ((A) and (C)) and *tll*^*g*^ ((B) and (D)) blastoderm embryos carrying the eve stripe 4+6 enhancer ((A) and (B)), or the eve stripe 3+7 enhancer ((C) and (D)) fused to *lacZ*, were stained for *lacZ* (purple), and *eve* mRNA (red). Arrows in (B) and (D) point at the late stripe-7-like expression domain in mutants. Lateral views. Anterior is to the left, dorsal is up.

We corroborated the identity of the most posterior stripe by driving *lacZ* expression in *tll*^*g*^ embryos under the control of the *eve* stripe 3+7 and 4+6 enhancers (Sackerson et al., 1999; Small et al., 1996). Expression from the 4+6 enhancer overlaps the most posterior stripe in mutants expressing six Eve stripes only (not shown), and matches the broadened penultimate stripe in those embryos expressing at least a partial 7th stripe (Fig. 3A and B). In contrast, we never observed expression (other than stripe 3) driven from the 3+7 enhancer in mutant embryos showing either 6 or 7 Eve stripes (Fig. 3C and D). This demonstrates that the missing posterior stripe is indeed stripe 7, while the identity of the late 7th stripe in mutants remains somewhat ambiguous. Our results indicate that this stripe is not driven by the 3+7 element. However, we may be missing weak posterior expression due to a lack of sensitivity in our methods, since the expression of stripe 7 driven by the 3+7 element in wild-type embryos is very weak (see, for example, Fig. 3A in Small et al., 1996).

**Fig. 4:**
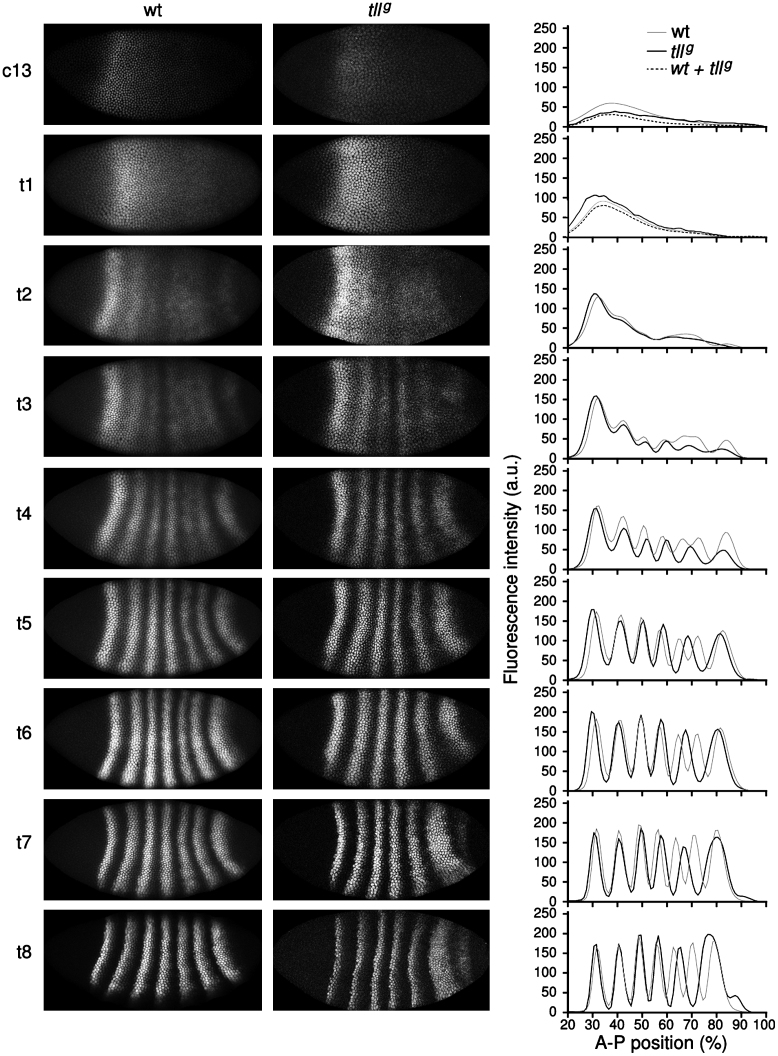
Comparison of Eve protein expression patterns between *tll*^*g*^ and wild-type embryos. Representative embryo images showing Eve protein expression in wild-type (left column) and *tll*^*g*^ embryos (middle column) are shown for each time class in c14A (t1–8). Lateral views; anterior is to the left, dorsal up. The right column shows corresponding integrated (averaged) expression patterns for each time class. Mutant data in black, wild-type data in grey. Vertical axes as in Fig. 2. For the horizontal axes, the entire Eve expressing region between 20 and 100% A–P position is shown (where 0% is the anterior pole). See the Materials and methods section for details on data quantification and time classification.

**Fig. 5:**
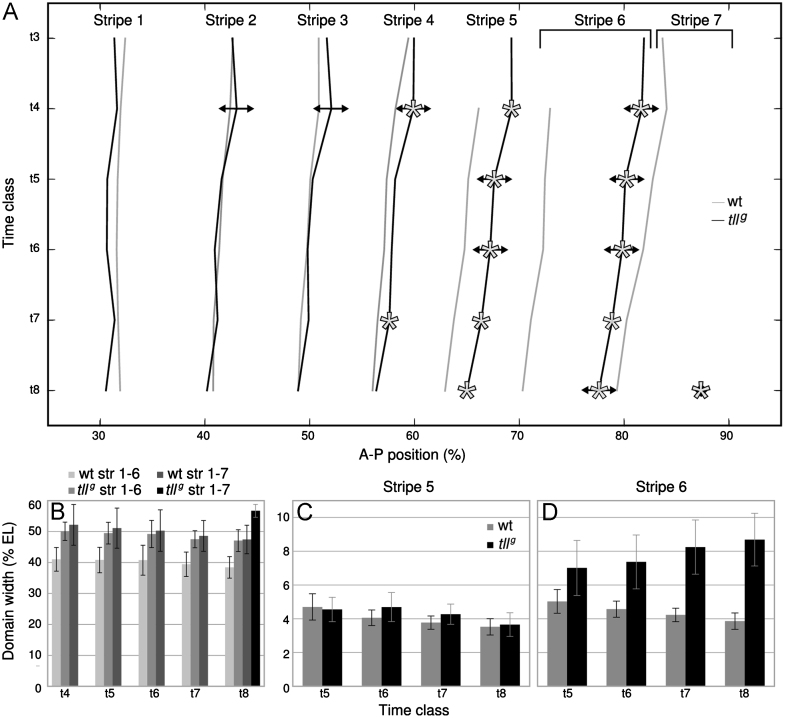
Eve protein expression dynamics and variability in *tll*^*g*^ compared to wild-type embryos. (A) A time-space plot showing average Eve stripe peak position from t3 to t8. Mutant data in black, wild-type in grey. Lines represent linear interpolation between measured time points. Vertical axis indicates time (increasing downwards); horizontal axis indicates percent A–P position (where 0% is the anterior pole). The region between 25 and 95% A–P position is shown. Stars indicate significantly shifted average peak position in *tll*^*g*^ compared to wild-type. Two-sided-arrows indicate significantly increased variance in peak position in *tll*^*g*^ compared to wild-type embryos. Increased variability and posterior displacement of all stripes in t4 may indicate an artefact in our data set. See the Materials and methods section for details on measurements of domain positions. (B) Shows the width of the entire region expressing Eve protein (in % egg length, EL) measured from the peak of stripe 1 to the peak of stripe 6 (*tll*^*g*^) or 7 (wild-type) across time classes in c14A. ((C) and (D)) Show the widths (in %EL) of Eve stripe 5 (C) and 6 (D) across time classes in c14A in wild-type versus *tll*^*g*^ embryos. In all panels, mutant data are shown in black, wild-type data in grey. Error bars represent one standard deviation from the mean.

Next, we analyzed the expression dynamics of the Eve protein in *tll*^*g*^ embryos in detail, and compared it to Eve expression in wild-type embryos (Fig. 4; see Surkova et al., 2008a, for a thorough characterisation of the wild-type pattern). In early time classes c13 and c14A-t1, there are only very subtle differences between integrated Eve patterns in wild-type versus *tll* mutant embryos (early embryos are difficult to distinguish based on Eve alone and were genotyped based on Tll staining, see the Materials and methods section and Table S1). The first clear difference between mutant and wild-type can be detected in t2: whereas Eve stripe 7 appears in wild-type embryos at this stage, it is completely absent in embryos lacking *tll*. In t3 mutant embryos, a 6th stripe appears close to or at the position of wild-type stripe 7 (see Table S2). This stripe forms in a way similar to stripe 6 in wild-type embryos, by splitting from a domain that is equivalent to the stripe 4–6 domain in the wild-type background (Surkova et al., 2008a). However, the mutant stripe 4–6 domain is less pronounced and shorter-lived than that in wild-type: at t4, stripes 5 and 6 are already clearly separated in the mutant, while they are still joined in wild-type. During t5 and t6, stripes 1–6 become fully formed in both mutant and wild-type backgrounds: they are now clearly separated, their boundaries sharpen, and expression intensity increases over time. While the position of stripes 1–3 are almost identical in wild-type and *tll* mutants (through all time classes), stripes 4–6 are located further towards the posterior in the mutant background. While this posterior dislocation is very subtle in the case of stripe 4, it is quite substantial for stripes 5 and 6 (see Fig. 5A). Statistical tests on our data set show that this more posterior location is significant at t4 and t7 for stripe 4, and from t4 to t8 for the more posterior stripes (Fig. 5A; Table S2). In t7 and t8 a small additional expression domain posterior of the 6th is observed in the integrated data, which reflects the appearance of a delayed 7th stripe in some of the mutant embryos (see below). Note that even in those mutant embryos exhibiting 7 Eve stripes, the pattern never resembles the wild-type.

**Supplementary Fig. S1:**
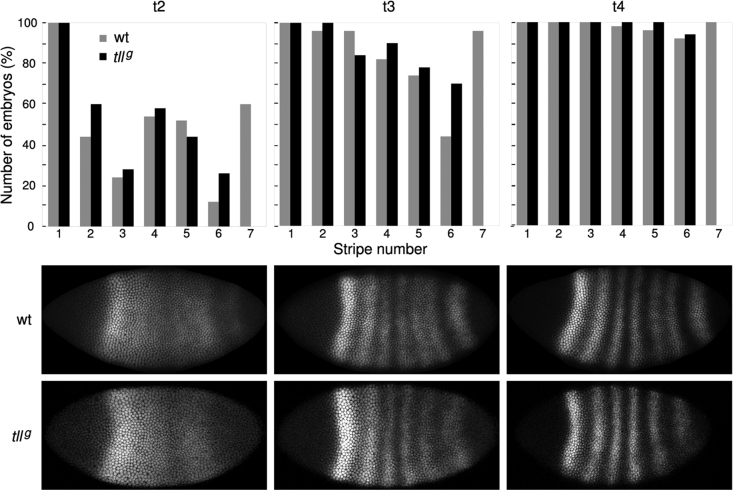
**The timing and order of Eve stripe formation differs in*****tll***^***g***^**compared to wild-type embryos.** Graphs show the percentage of embryos in our data set in which particular stripes have formed at time class t2 (first column), t3 (second column), and t4 (fourth column). Samples represent 50 randomly chosen wild-type and *tll*^*g*^ embryos from our data sets. Each bar represents a particular stripe as indicated on the horizontal axis. Mutant data are shown in black, wild-type data in grey. Embryo images show representative Eve protein expression patterns in wild-type (first) and *tll*^*g*^ (second row) at t2, t3 and t4. Lateral views; anterior is to the left, dorsal is up.

Comparing stripe formation in both genotypes reveals that Eve stripe 6 forms earlier in *tll* mutants than in wild-type embryos: in t2 and t3 there is a higher percentage of mutant embryos that have a 6th stripe compared to wild-type (Fig. S1). This discrepancy between wild-type and *tll* mutants is corrected by t4 as both show properly formed 6th stripes at this and later time points during the blastoderm stage.

**Supplementary Fig. S2:**
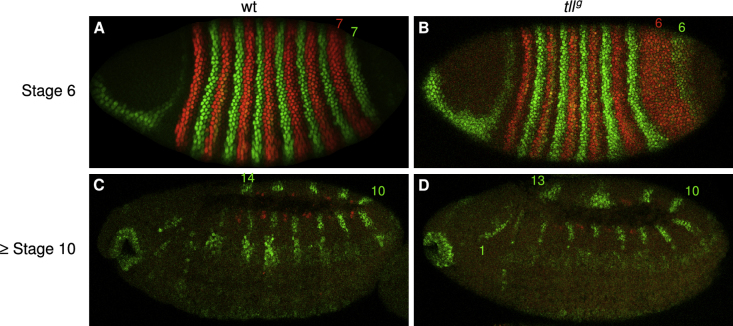
**Posterior defects in pair-rule gene expression persist during and after gastrulation.** This figure shows wild-type ((A) and (C)) and *tll*^*g*^ embryos ((B) and (D)) stained for Eve and Odd protein at Bownes’ stage 6 (gastrulation; (A) and (B)) and stage 10 or older (extended germ-band; (C) and (D)). Antibody stains are shown for Eve (red) and Odd (green). Persistent patterning defects and missing stripes are observed at the posterior end of the germ-band in *tll* mutant embryos ((B) and (D)). Stripe numbers are indicated for clarity.

**Supplementary Fig. S3:**
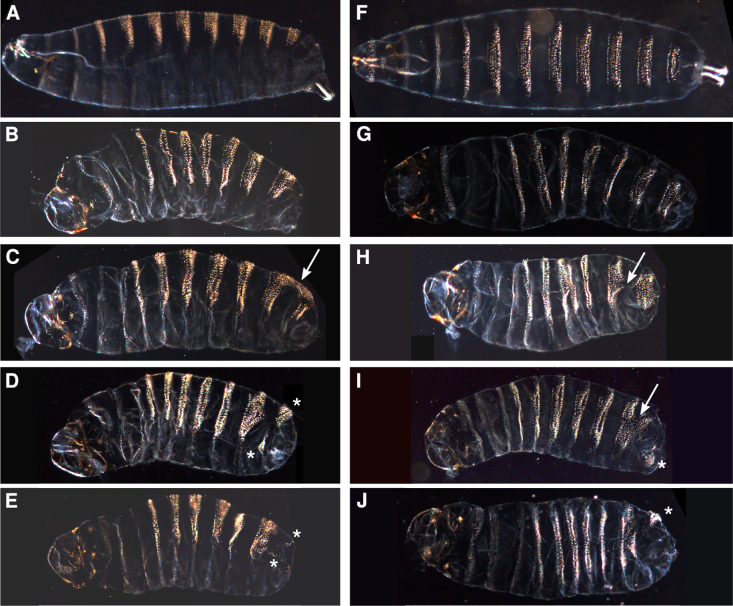
**Variable cuticle phenotypes at late stages of embryogenesis reflect variability in posterior segmentation gene expression at the blastoderm stage.** Cuticle preparations of unhatched first-instar larvae are shown for wild-type (A,F) and homozygous *tll* mutant embryos ((B)–(E), and (G)–(J)) in lateral ((A)–(E)), and dorsal orientation ((F)–(J)). All *tll* mutants are missing the A8 denticle belt as well as the telson. Additional defects include fused (arrows in (C), (H), and (I)) and incomplete denticle belts or small denticle patches (asterisks in (D), (E), (I), and (J)).

Similarly, could it be that the lack of stripe 7 expression in mutants, which appears in a subset of embryos at t7 and t8, is also due to a temporal delay and is corrected at a subsequent stage of development? In other words, will mutant embryos eventually express a complete 7-stripe pair-rule pattern? To investigate this possibility, we analyzed protein expression patterns of Eve and Odd-skipped (Odd) in *tll* mutant embryos during and after gastrulation. Our results indicate that the posterior defects we observe at the blastoderm stage persist throughout development. We observe disruption of posterior pair-rule gene expression in *tll* mutant embryos up to the extended germband stage at which the Eve segmentation pattern disappears (Fig. S2). Furthermore, all *tll* mutants – regardless of how many Eve stripes they show at gastrulation time – exhibit morphological defects in posterior abdominal segments and structures of the telson (Fig. S3; Jürgens et al., 1984; Pignoni et al., 1990, 1992; Strecker et al., 1986, 1988a,b).

Surkova et al. (2008a) showed that wild-type Eve stripes shift to the anterior from t3 to t8. In *tll* mutants, the anterior Eve stripes shift over a similar amount of nuclei, whereas the posterior stripes, mainly stripes 5 and 6, shift slightly more than the equivalent stripes in wild-type (Fig. 5A, Table S3). Interestingly, stripe 6 in mutants shifts as much as stripe 7 in wild-type, suggesting that stripe position, not identity, may be relevant for the extent of the shift (see Discussion).

Assayed across the entire blastoderm stage, most *tll* mutants only express six rather than seven Eve stripes. Nevertheless, the total width of the Eve expression domain (measured by the range from the peak of stripe 1 to the peak of stripe 7 in wild-type embryos, and to the peak of stripe 6 in mutant embryos) remains very similar between wild-type and mutants up to t6, before the appearance of stripe 7 in a subset of mutant embryos (Fig. 5A and B). This width is approximately 50 nuclei (Fig. 5B). In mutant embryos that show a 7th stripe, this stripe appears more posterior than the equivalent stripe in wild-type and hence the total expression domain expands: 57 nuclei in *tll* compared to 47 nuclei in wild-type embryos at t8 (Fig. 5B). Measuring the width of individual stripes in *tll* mutants reveals that the 5th (t6, t7) and 6th stripe (t5–8) are wider than the respective wild-type stripes (Fig. 5C and D; see Table S4 for statistical significance). This effect is small for stripe 5 (<1 nucleus), while it is considerable for stripe 6, which is ∼2 nuclei wider than wild-type at t4, increasing to a difference in width of almost 5 nuclei at t8 (Table S4).

### Increased variability of Eve expression in *tll* mutants

**Fig. 6:**
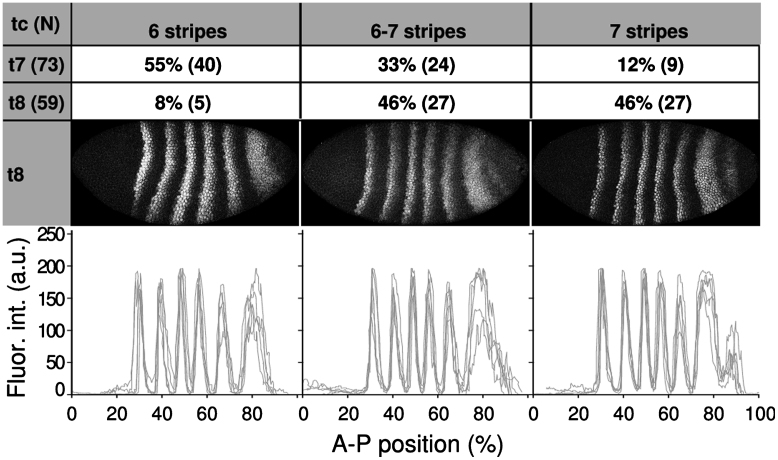
Eve protein expression is highly variable in *tll*^*g*^ embryos during the late blastoderm stage. The two upper rows show percentages of embryos in our data set that show 6 stripes, 7 stripes, or an intermediate pattern in t7 (first row) and t8 (second row). *N* indicates total number of embryos in each time class. Embryo images show Eve protein expression in individual embryos belonging to the same time class demonstrating the variability of Eve expression during t8. Lateral views; anterior is to the left, dorsal is up. See main text for detailed description of patterns and embryo classification criteria. Graphs (bottom) show representative expression profiles (background removed, not registered) from 5 individual mutant embryos (grey lines) belonging to the same time class, and exhibiting the same number of Eve stripes.

As we have mentioned above, a subset of mutant embryos form a delayed 7th stripe of Eve expression. Based on this, we can subdivide our data set into three classes of embryos showing qualitatively different kinds of gene expression towards the end of the blastoderm stage (at t7 and t8, right before gastrulation). The three classes are defined as follows: (1) embryos that show 6 stripes, (2) embryos that show 6 stripes plus a partial 7th stripe (not fully separated from the 6th or not spanning the entire dorso–ventral, or D–V axis), and (3) embryos that show 7 fully formed Eve stripes (Fig. 6). The 7th stripe appears progressively: at t7, the majority of embryos (55%) show 6 stripes only, about a third (33%) show partial expression, and only a small number (12%) show a completely established stripe 7 (Fig. 6). In contrast, a large majority of mutant embryos (92%) exhibit a partially or fully formed 7th stripe at t8 (Fig. 6). This indicates a significant increase in embryo-to-embryo variability in the posterior region of the embryo: while some embryos initiate stripe 7 relatively early (t7), others only start expressing it at t8. The rest either initiate it after gastrulation, or never show any stripe 7 expression at all (see above, and Fig. S2).

In addition, embryo-to-embryo variability in stripe 7 expression is reflected by increased variability in peak and boundary positions for stripes 5 and 6 among mutants (Fig. 6, bottom row). For stripe 5, this is statistically significant at t5 and t6; for stripe 6, from t3 to t8 (with the potentially artifactual exception of t7; Fig. 5A, Table S5). Moreover, there is a significant increase in the variability of the width of Eve stripe 6 in *tll* mutants (Fig. 5D, Table S6).

Finally, variability in gene expression patterns during the blastoderm is reflected by variation of the mutant phenotype at later stages of development. Cuticle preparations of unhatched first-instar larvae show significant variation in the extent of affected or missing posterior denticle belts and structures. While none of the embryos show eight abdominal segments, and all are missing the telson, some embryos show incomplete denticle belts and small denticle patches (asterisks in Fig. S3D, E, I and J), or fused denticle belts (arrows in Fig. S3C, H and I) in A6 and A7. Although this evidence does not provide a direct link between gene expression and morphological variability, it strongly suggests that the failure of canalisation observed during early embryogenesis persists throughout development.

### Maternal and gap gene expression in *tll* mutants

**Supplementary Fig. S4:**
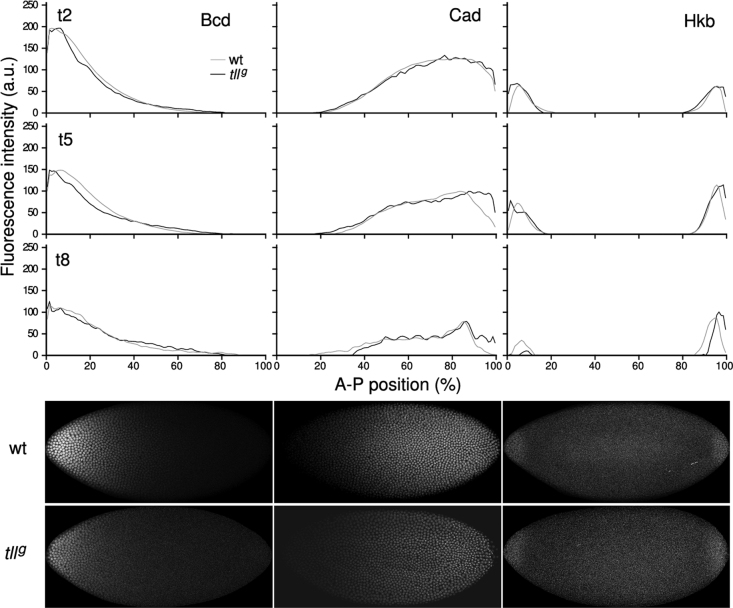
**Protein expression patterns of Bcd, Cad and Hkb are similar in*****tll***^***g***^**and wild-type embryos.** Graphs show integrated (averaged) expression patterns for Bcd (first), Cad (second), and Hkb (third column) in *tll*^*g*^ mutants (black) compared to wild-type (grey) at t2 (first), t5 (second) and t8 (third row). Vertical axes show fluorescence intensity in arbitrary units ranging from 0 to 255. Horizontal axes represent *A*–*P* position in percent (where 0% is the anterior pole). The entire length of the embryo (0–100% *A*–*P* position) is shown. Embryo images show representative protein expression patterns for *tll*^*g*^ (first) and wild-type (second row) at time class t2 stained against Bcd (first), Cad (second) and Hkb (third column). Lateral views; anterior is to the left, dorsal is up. See Materials and methods section for details on data quantification and time classification.

In order to understand the altered and more variable nature of Eve dynamics, we analyzed the expression of its upstream regulators, the maternal co-ordinate and gap genes, in *tll*^*g*^ mutants. The expression profiles of the maternal co-ordinate proteins Bcd and Cad are the same in *tll* compared to wild-type embryos (Fig. S4). The only exception is a delay in the retraction of the (zygotic) posterior Cad expression domain from the posterior pole: in wild-type embryos, the posterior Cad domain retracts at t4, whereas in *tll*^*g*^ embryos, it only retracts at t8. This delay had not been detected in the hypomorphic *tll*^*1*^ allele (Reinitz and Levine, 1990). Similarly, expression of the terminal gap protein Hkb is essentially unaltered in *tll* mutants (there may be a very slight shift in the position of its posterior domain at late stages, but the effect is subtle; Fig. S4).

**Fig. 7:**
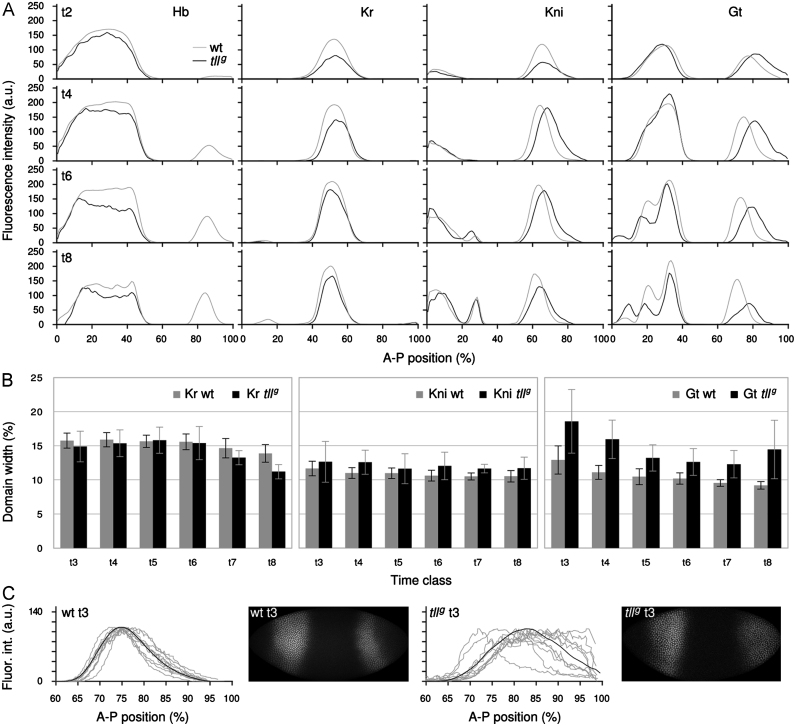
Dynamics and variability of gap gene expression in *tll*^*g*^ versus wild-type embryos. (A) Integrated (averaged) expression patterns of Hb (first column), Kr (second column), Kni (third column), and Gt (fourth column) in *tll*^*g*^ mutants (black) compared to wild-type (grey) are shown at t2 (first row), t4 (second row), t6 (third row) and t8 (fourth row). Plot axes as in Fig. 2. The entire length of the embryo (0–100% A–P position) is shown. (B) Domain widths (in %EL) of the central Kr, the abdominal Kni, and the posterior Gt domain across c14A time classes (t3–t8) in wild-type versus *tll*^*g*^ embryos. In all panels in (B), mutant data are shown in black, wild-type data in grey. Error bars represent one standard deviation from the mean. (C) Variability in the expression of the posterior Gt domain in *tll*^*g*^ embryos at t3. Images show Gt protein expression in a wild-type (left), and a *tll*^*g*^ mutant embryo (right). Graphs show expression profiles from 10 randomly chosen individual embryos (grey lines) together with integrated expression data (black) at time class t3 for wild-type (left), and *tll*^*g*^ embryos (right graph; data not registered, but background removed).

Just as in the case of Eve stripe positions, positions of mutant gap gene protein domains differ more significantly towards the posterior of the embryo (Fig. 7A, Table S7). We confirmed the observation of Casanova (1990) that there is no posterior Hb domain in *tll* null mutants, whereas the anterior Hb domain is very similar to wild-type. Neither is the position or width of the central domain of Kr affected (Fig. 7A and B; Gaul and Jäckle, 1987; Reinitz and Levine, 1990). In contrast, both the abdominal Kni domain and the posterior Gt domain are located further towards the posterior pole in mutants compared to wild-type (Fig. 7A, Table S7; Eldon and Pirrotta, 1991; Kraut and Levine, 1991; Pankratz et al., 1989). This effect is very subtle in the case of Kni (Fig. 7A): we observe a slight posterior dislocation, but no significant expansion of this domain (Fig. 7B). Gt, on the other hand, shows a pronounced expansion towards the posterior, which is caused by delayed retraction of the domain from the pole, which happens at t1 in wild-type, but only between t2 and t3 in most *tll* mutants (Fig. 7A and B). In agreement with previously published observations, it is difficult to detect any significant defects in the anterior expression domains of both Kni and Gt (Eldon and Pirrotta, 1991; Rothe et al., 1994).

Both Kr and Kni show a potential delay in the onset of expression: in wild-type, Kni protein can first be detected at c13 (Surkova et al., 2008a), whereas in *tll*^*g*^ embryos, the Kni protein only appears at t1 (data not shown; it is still significantly weaker than wild-type at t2, see Fig. 7A). Similarly, Kr expression seems slightly delayed with no or very weak expression in mutant c13 embryos versus strong c13 expression in wild-type (data not shown; again, expression is still weaker at t2, Fig. 7A).

**Supplementary Fig. S5:**
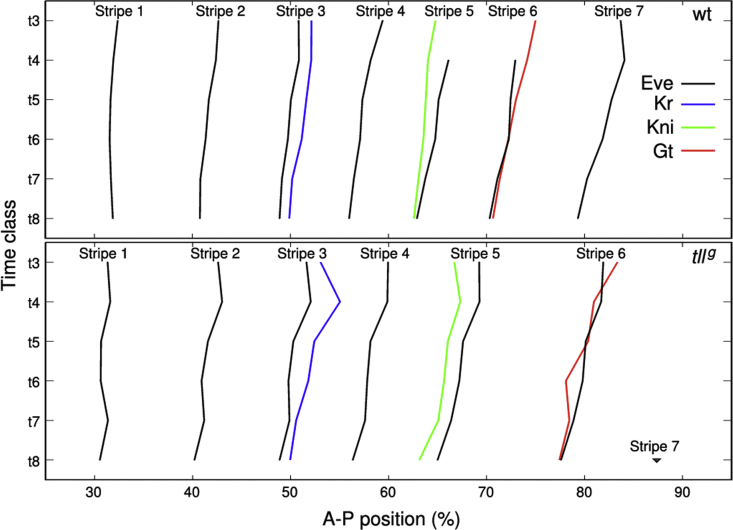
**Relative positions and temporal shifts of Eve stripes and gap domains are maintained in wild-type versus*****tll***^***g***^**embryos.** Time-space plots show average Eve stripe and gap domain peak positions from t3 to t8 in wild-type (top) and *tll*^*g*^ mutants (bottom panel). Eve is shown in black, gap genes in colour. Lines represent linear interpolation between measured time points. Vertical axes indicate time (flowing downwards); horizontal axes indicate percent *A*–*P* position (where 0% is the anterior pole). The region between 25 and 95% *A*–*P* position is shown. See Materials and methods section for details on measurements of domain positions.

In wild-type embryos, Eve stripes follow the dynamic anterior shifts of gap domains over time (Jaeger et al., 2004; Surkova et al., 2008a). We observe similar dynamics in *tll* mutants: shifts in Eve stripes are very similar to those observed for gap domains in the same region (Fig. S5). This implies that the more posterior gap domains (abdominal Kni and posterior Gt) shift more in mutants than in wild-type embryos (Fig. S5, Table S8). The widths of gap domains also differ towards the posterior of the embryo. In particular, the posterior Gt domain is significantly wider in *tll* mutants compared to wild-type (Fig. 7B, Table S9).

**Supplementary Fig. S6:**
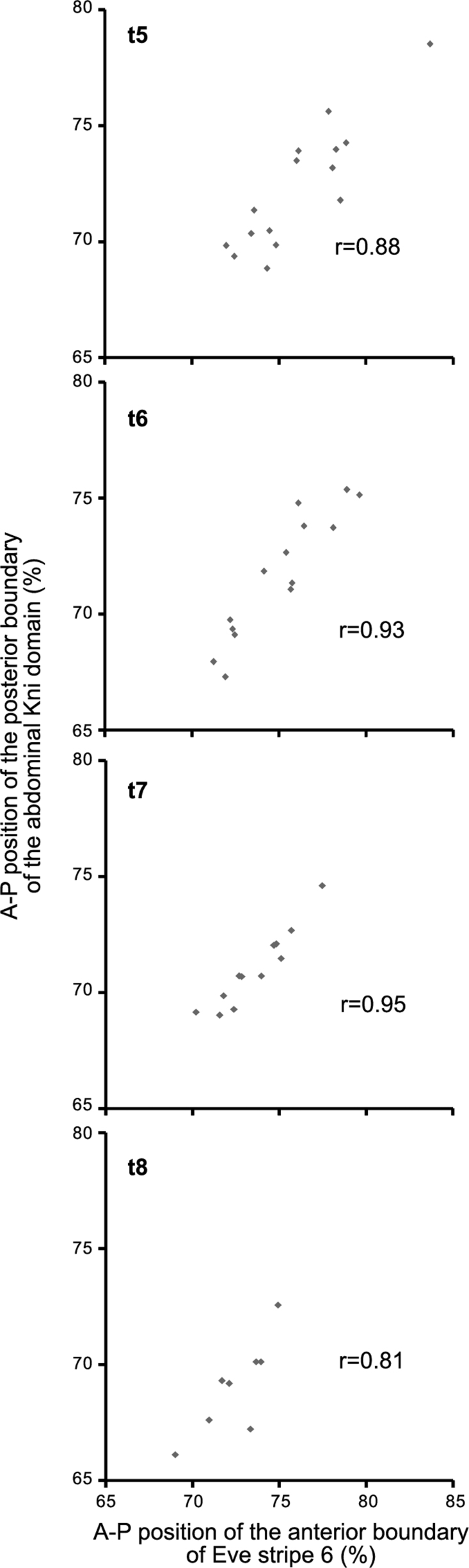
**Co-ordinated dynamic shifts of the abdominal Kni domain and Eve stripe 6 in*****tll*****mutants.** Scatter plots show strong correlations between the position of the anterior boundary of Eve stripe 6 (*x*-axes) and the posterior boundary of abdominal Kni (*y*-axes) at time classes t5–8, when expression features are clearly identifiable. *r* are Pearson correlation coefficients.

To corroborate the link between shifts in gap domain and Eve stripe positions, we examined the correlation between the position of the posterior boundary of abdominal Kni, and the anterior boundary of Eve stripe 6. It has been shown that Kni is involved in positioning this Eve boundary (Frasch and Levine, 1987; Clyde et al., 2003). We find a very strong correlation at all time classes for which it was possible to accurately measure the relevant borders (Fig. S6). This provides further evidence that the dynamics of Eve expression in *tll* mutants is largely dependent on shifting gap domain positions, as in the case in the wild-type background (Surkova et al., 2008a).

### Increased variability in mutant gap gene expression

Finally, we investigated embryo-to-embryo variability in mutant gap gene expression. This signal is more difficult to confirm by statistical testing due to the lower number of embryos stained against gap proteins compared to Eve in our data set (see Discussion). Standard deviations for peak and boundary positions of the central Kr, abdominal Kni, and posterior Gt domains are generally slightly higher in *tll* mutants than in wild-type embryos (Table S10). This effect is particularly strong for early (t3) patterns of Kni and Gt. It is very striking when looking at individual profiles and embryo images (Fig. 7C): the posterior Gt domain in *tll* mutants ranges from wild-type width to expanded posterior expression reaching all the way to the posterior pole. At later stages, the abdominal Kni domain shows gradually diminishing variability over time, whereas the posterior Gt domain suddenly becomes much more precise at t4. Similar trends can be observed when measuring gap domain width (Table S11).

## Discussion

In this study, we measured the timing and position of gap domains, and the 7 stripes of Eve in *tll* mutant embryos at high accuracy and spatio-temporal resolution. We compared the average position and variability of domain peaks and boundaries, the width of each domain, as well as the extent of anterior domain shifts between mutants and wild-type embryos.

### Co-ordinated fate map changes in *tll* mutants

Our quantitative analysis of mutant segmentation gene expression confirms and extends previously published evidence based on qualitative observations: (1) Assayed across the entire duration of cleavage cycle 14A (c14A), a large majority of *tll* mutants exhibit only 6 stripes of Eve expression, which span approximately the same region of the embryo as the 7 stripes observed in wild-type (Figs. 2, 4 and 5B; Frasch and Levine, 1987). Our results corroborate previous evidence that the missing stripe is stripe 7 (Fig. 3; Fujioka et al., 1999). A small percentage of c14A mutant embryos do express a 7th stripe, which appears posterior of the corresponding wild-type stripe (Fig. 5A and B, Fig. 6; Frasch and Levine, 1987). (2) The posterior Hb domain is missing (or so weak it is not detectable) in *tll* mutants (Figs. 2 and 7; Brönner and Jäckle, 1991; Casanova, 1990; Margolis et al., 1995; Reinitz and Levine, 1990). (3) The posterior domain of Gt is expanded and shifted towards the posterior, due to its delayed retraction from the posterior pole in the mutant background (Figs. 2 and 7A and B; Eldon and Pirrotta, 1991; Kraut and Levine, 1991). (4) Similarly, the abdominal Kni domain is located at a more posterior position in *tll* mutants compared to wild-type (Figs. 2 and 7A and B). However, this effect is subtle, and we have not been able to measure a significant increase in the width of this domain as observed for *kni* mRNA by Pankratz et al. (1989). (5) The anterior domain of Hb and the central domain of Kr are not affected in *tll* mutants (Fig. 7A; Casanova, 1990; Gaul and Jäckle, 1987; Reinitz and Levine, 1990). Neither are the anterior domains of Gt and Kni (Fig. 7A; Eldon and Pirrotta, 1991; Rothe et al., 1994), nor the two terminal domains of Hkb and the maternal expression patterns of Bcd and Cad (Fig. S4; Brönner and Jäckle, 1996; Reinitz and Levine, 1990).

In addition to the expression patterns analyzed here, there is evidence that the posterior domain of the gap-like gene *nubbin (nub*; also called *pdm1*) retracts late from the posterior pole (Cockerill et al., 1993), and that the posterior-most stripes of the pair-rule genes *fushi tarazu (ftz)*, *hairy (h)*, and *odd-skipped (odd)* are missing in *tll* mutants (Casanova, 1990; Mahoney and Lengyel, 1987; Reinitz and Levine, 1990; Riddihough and Ish-Horowicz, 1991; La Rosée et al., 1997, and this study). Furthermore, Hox gene expression is affected in an analogous way: *Antennapedia (Antp)* expands towards the posterior, and *AbdominalB (AbdB)* is absent in *tll* mutants (Casanova, 1990; Reinitz and Levine, 1990).

Positional information in the *Drosophila* blastoderm depends on the position and dynamics of segmentation gene expression boundaries (reviewed in Jaeger and Reinitz, 2006). Therefore, the delayed, shifted and/or missing posterior expression domains described above provide a molecular explanation for the fact that the fate map of the embryo is distorted and stretched towards the posterior pole in *tll* mutants, which leads to the observed loss of terminal structures (Strecker et al., 1986).

This distortion of the fate map appears to occur in a co-ordinated manner across expression patterns of different segmentation genes. Our analysis reveals that displacement of domains in mutants compared to wild-type embryos is similar at equivalent positions along the A–P axis (Fig. 2, Fig. S5). Overall, this results in a preserved relative order of domains with respect to each other. For instance, the 5th stripe of Eve, although shifted to the posterior, is still centred on the abdominal domain of Kni, and Eve stripe 6 coincides with the posterior Gt domain, bordering the abdominal Kni domain in *tll* mutants as it does in wild-type (Fig. 2, Figs. S4 and S6). In the case of Eve and Gt, such co-ordinated displacement has been described previously by Kraut and Levine (1991). Note that this observation is neither obvious nor trivial. In contrast to our results, Surkova et al. (2013) report that, in a *Kr* mutant background, the posterior domain of *gt* exhibits drastic dynamic changes in its position with regard to other gap domains. While early *gt* expression is located at the posterior pole (similar to wild-type), a large anterior shift occurs over time such that *gt* expression almost completely coincides with that of *kni* at late stages in these mutants. This results in a substantially reordered relative arrangement of the domains in mutants compared to wild-type. In this case, the response to the mutation is gene- or domain-specific.

In the case of *tll*, however, the situation is different. We observe no gene- or domain-specific effects. The order of expression domains along the A–P axis in *tll* mutants is preserved compared to wild-type, which requires co-ordinated changes in gene expression dynamics resulting in a corresponding co-ordinated displacement of domain positions. This has specific consequences for the expected extent of dynamic shifts in domain positions over time. When comparing mutant and wild-type embryos, we would expect these shifts to be similar at equivalent A–P positions, rather than similar across equivalent expression domains. This is exactly what we observe. For example, the posterior Gt domain shifts by 4.34% egg length (EL) in wild-type, but by 5.92%EL in mutants, according to its more posterior position (Fig. S5; Table S8). Similarly, Eve stripe 6 shows a shift of 4.16%EL between t3 and t8 in mutants, which is much closer to the shift of stripe 7 (4.36%EL) than that of stripe 6 (2.62%EL) in wild-type (Fig. 5A; Table S3). This dependence on position rather than domain identity is not obvious, especially in the case of Eve, since stripes 6 and 7 are regulated by different *cis*-regulatory elements implementing different regulatory mechanisms (Fujioka et al., 1999; Harding et al., 1989; Sackerson et al., 1999; Small et al., 1996).

One last aspect of the mutant fate map deserves attention: the influence of *tll* reaches much further than the extent of its expression domain. While patterning defects in *kni* and *eve* expression can be observed from about 55–60% A–P position all the way to the posterior pole (with increasing severity of defects towards the terminal region; Frasch and Levine, 1987), the expression of *tll* is confined to the posterior-most 20% of the embryo (Figs. 1 and 5–7, Tables S2, S4, S7 and S9). It is not clear how these extended expression defects can be accounted for. One possibility is that we simply cannot detect low, but functionally important, levels of Tll expression in the middle of the wild-type embryo. Another explanation could be that the regulatory effect of Tll may not be entirely cell-autonomous. Segmentation gene products can diffuse between nuclei in the syncytial embryo, since cellularisation remains incomplete until very close to gastrulation time (Foe and Alberts, 1983). Such a cell-non-autonomous effect, however, would be unexpected, since the regulatory mechanism underlying gap domain shifts, which could convey an extended influence of Tll, has been shown to work in an entirely cell-specific manner (Jaeger et al., 2004; Manu et al., 2009b; see also below). Further studies will be required to resolve this apparent paradox.

### Dynamics of mutant gene regulation and expression

The quantitative analysis presented here expands our understanding of gene expression and regulation in *tll* mutants in several important ways. The first new insight concerns the timing of segmentation gene expression. In those mutant embryos that do show expression of a 7th Eve stripe, its formation is severely delayed from early c14A (t2/t3) in wild-type to just before the onset of gastrulation (t7/t8) or even later in the mutant background (Figs. 2, 4, 5A, 6, S2). This explains the small percentage of embryos that were observed to express 7 stripes in an earlier study (Frasch and Levine, 1987).

Second, our analysis provides new insights into the regulatory mechanism by which gap domains shift towards the anterior of the embryo over time (Jaeger et al., 2004; Surkova et al., 2008a). This mechanism depends on asymmetric repression with posterior dominance between overlapping gap gene domains: Hb represses *gt*, Gt represses *kni*, Kni represses *Kr*, but not vice versa (Jaeger and Reinitz, 2006; Jaeger et al., 2004). If these repressive effects are assumed to work as a sequential cascade, from posterior to anterior (i.e., from *hb* to *gt* to *kni* to *Kr*), then this mechanism depends crucially on the presence of a posterior Hb domain (Jaeger and Reinitz, 2006). In other words, this interpretation suggests that the formation of the posterior Hb domain during c14A displaces and shifts the other gap domains towards the anterior, predicting that shifts should be reduced or absent in *tll* mutants. Our results provide conclusive evidence against such a sequential mechanism.

Gap domain shifts still occur in *tll* mutants that have no posterior Hb. Shifts are not triggered by the appearance of this domain, but occur in a simultaneous and autonomous manner. Basically, the observed spatial shifts are produced by each nucleus in the posterior embryo cycling through the expression of a succession of gap genes: *Kr*, then *kni*, then *gt*, then *hb*. Depending on their position, nuclei enter this cascade at different times. For instance, a nucleus in the central region will start expressing *Kr* then switch to *kni*, while a more posterior nucleus may start expressing *kni*, which gets replaced by *gt*, which in turn is superseded by *hb* through the asymmetric repressive interactions described above. Our results are consistent with the idea that initiation of this process is independent of posterior Hb, although premature truncation of the cascade occurs in *tll* mutants. This interpretation is in complete agreement with a theoretical analysis of gap gene regulation, which predicts a cell-autonomous shift mechanism independent of the influence of Tll (Manu et al., 2009b).

### Variability of mutant gene expression

The final, and most important result of our analysis concerns the increased variability of gene expression in *tll* mutant embryos. This affects both the timing of expression, as well as the position and width of posterior gap and pair-rule domains.

The position and width of the 6th Eve stripe are significantly more variable in *tll* mutants compared to wild-type (Fig. 5, Tables S5 and S6). Similarly, domain positions and widths of both the abdominal Kni and the posterior Gt domain vary considerably from embryo to embryo in mutants, while they are positioned very precisely and reproducibly in wild-type embryos at the late blastoderm stage (Fig. 7B and C, Tables S10 and S11; Surkova et al., 2008a).

Note that, in contrast to Eve, increased variability in gap gene expression was not tested for statistical significance due to the smaller number of embryos in the gap gene data set (Table S1). Nevertheless, we believe the effect to be real: strongly increased variability in domain position is clearly visible when comparing individual expression profiles between wild-type and *tll* mutant embryos (Fig. 7C).

Another caveat is that the detected increase in positional variability could be due to the increased extent of domain shifts in *tll* mutants (Tables S4, S9). Larger (and hence faster) dynamic shifts imply that domain boundaries can vary between embryos simply because they were measured at a slightly different stage that cannot be discriminated by our time-classification scheme. However, there are two reasons why this is unlikely to account for all the measured increase in positional variability. First, domains shift only by about 1–2%EL more in mutants versus wild-type across five time classes (about 30–35 min) from t3 to t8 (Tables S4 and S9). This difference is much smaller than most of the measured differences in positional variances within a time class of about 6–7 min (Tables S5 and S10). Second, it is unlikely that this effect can account for the increased variability observed in domain widths (Tables S6 and S11). This is because positions of anterior and posterior domain boundaries are correlated as both shift in the same direction over time.

Increased variability in Eve stripe 7 concerns mainly temporal rather than spatial aspects of gene expression. The timing of stripe formation differs greatly between embryos: while some *tll* mutants start expressing Eve stripe 7 at t7, others initiate it at t8, and a sizeable proportion never express this stripe at all during c14A (Fig. 6). Furthermore, some embryos show clear separation between stripes 6 and 7, while others show fused or incompletely separated stripes. Lastly, expression of stripe 7 does not span the entire D–V axis in many *tll* mutant embryos. This leads to qualitatively different patterns of gap and pair-rule gene expression in different embryos at the late blastoderm stage, a phenomenon which is never observed in a wild-type background (Surkova et al., 2008a).

The regulatory mechanism underlying the observed variability in Eve stripe 7 expression remains unknown. However, our evidence suggests a possible scenario. We observe a trend in our data set towards greater width of Eve stripe 6 in embryos expressing a 7th stripe before gastrulation (data not shown). Moreover, we fail to detect expression of the 3+7 element in the region of stripe 7 in *tll* mutants at t7/8 (Fig. 3D). Taken together, this can be interpreted in the following way: it may be that stripe 7 expression in *tll* mutants is solely driven by the late (or auto-regulatory) enhancer element of eve (Fujioka et al., 1995, 2002; Goto et al., 1989; Harding et al., 1989; Jiang et al., 1991). It has been shown earlier (in the context of stripes 5 and 6) that this element responds to a wide range of Eve concentrations (Fujioka et al., 1995). Therefore, stripe 7 expression could be triggered by the late element in mutant embryos showing a wide Eve stripe 6 – and hence an increased amount of Eve protein in the posterior region of the embryo – assuming that only these embryos reach the threshold level required for auto-activation. Amplification of variability by auto-regulation could explain the apparent increase in variability of Eve compared to gap genes (but not the source of the variability at the level of the gap gene system; see below). A rigorous investigation of this mechanism goes beyond the scope of this study, as it will require careful quantification of absolute Eve concentrations, and their effect on late-element expression in the relevant region of the embryo.

### Posterior loss of canalisation in *tll* mutants

Taken together, the increased positional and temporal variability in gene expression indicates significant de-canalisation of segmentation gene expression in the posterior of the embryo in *tll* mutants. Interestingly, de-canalisation also seems to be most severe in posterior regions of *Kr* and *kni* mutants, although these genes are expressed in or near the centre of the embryo (Surkova et al., 2013). In other words, deletion of *tll* (and certain other gap genes) seems to expose a vulnerability in the regulatory system which predominantly affects the posterior of the embryo. This vulnerability is hidden or buffered in the wild-type background. It renders the system sensitive to stochastic, genetic, and/or environmental fluctuations or perturbations leading to high embryo-to-embryo variability in posterior segmentation gene expression domains. This decrease in robustness is substantial considering the small amount of genetic and environmental variation expected to be present in our inbred strains of *Drosophila* under laboratory conditions. It is even more striking if we consider the fact that wild-type *Drosophila* embryos can tolerate large perturbations—such as heterozygous null mutations in any segmentation gene (see Introduction, and accompanying paper by Surkova et al., 2013).

The decrease in robustness in *tll* mutants is not only drastic, but also surprising, since it is not immediately obvious what is causing the vulnerability. It cannot depend on variable residual levels of Tll protein since we have used deletion strains that eliminate *tll* altogether. Moreover, Tll provides an entirely feed-forward regulatory input to the gap and pair-rule systems, since it is not itself regulated by other zygotic segmentation genes (Brönner and Jäckle, 1991; Brönner and Jäckle, 1996). Therefore, we cannot easily attribute the loss of robustness to the loss of a feedback control mechanism.

One possible explanation is a loss of redundancy (Wagner, 2005): regulatory inputs from the terminal gap genes Tll and Hkb to the gap gene system are at least partially redundant (Ashyraliyev et al., 2009; Brönner et al., 1994; Brönner and Jäckle, 1996; Weigel et al., 1990). A loss of this redundancy could cause increased sensitivity of posterior gap gene expression to variable levels of Hkb in *tll* mutant embryos. However, detected levels of variability in Hkb expression were rather limited compared to those observed for Kni and Gt expression (Fig. 7C, and data not shown). Therefore, alternative mechanisms, such as a loss of distributed robustness in the system (Wagner, 2005) may also play a role in this context.

Factors that uncover cryptic vulnerabilities in regulatory systems when mutated have been called genetic (or evolutionary) capacitors (Bergman and Siegal, 2003; Masel and Siegal, 2009). The classic example of such a genetic capacitor is Hsp90 (Queitsch et al., 2002; Rutherford and Lindquist, 1998). Hsp90 is a network hub, playing a central and privileged role in cellular regulation, since it is involved in the regulation of hundreds of different proteins involved in a large number of regulatory pathways (Rutherford et al., 2007). Furthermore, the work by Levy and Siegal (2008) on capacitors in yeast suggests that they are preferentially hub proteins in regulatory networks. Tll, on the other hand, is neither central nor remarkable in any other way, considering its role as a transcriptional regulator and member of the segmentation gene network. In fact, it is rather peripheral in this context, and provides a purely feed-forward and partially redundant regulatory input to the regulation of gap and pair-rule genes.

The fact that a regulator like Tll can play the role of genetic capacitor suggests that genetic capacitance may be a much more widespread phenomenon than previously thought. Our results provide further experimental evidence supporting this idea, which was first proposed by Bergman and Siegal (2003). These authors performed evolutionary simulations, which indicated that genetic capacitance may not be limited to specific factors (such as Hsp90) that play a central role in regulation.

Further insight into this problem requires a rigorous investigation of the dynamic regulatory mechanism responsible for the observed increase in gene expression variability. The data set presented in this paper provides a necessary and important first step towards such an investigation. It can be used for reverse-engineering the segmentation gene network in the presence and absence of Tll (see, for example Ashyraliyev et al., 2009; Jaeger et al., 2004; Manu et al., 2009a,b). This will result in dynamical models of the gap gene network, which can be exposed to sensitivity analysis with the aim to uncover the mechanistic causes of the observed breakdown in canalisation.

## Footnotes

Appendix A Supplementary data associated with this article can be found in the online version at doi:10.1016/j.ydbio.2013.01.010.
