# Erratum to “Quantitative dynamics and increased variability of segmentation gene expression in the *DrosophilaKrüppel* and *knirps* mutants” [Dev. Biol. 376 (2013) 99–112]

**DOI:** 10.1016/j.ydbio.2013.04.001

**Published:** 2013-05-01

**Authors:** Svetlana Surkova, Elena Golubkova, Manu, Lena Panok, Lyudmila Mamon, John Reinitz, Maria Samsonova

**Affiliations:** a Department of Computational Biology, Center for Advanced Studies, St. Petersburg State Polytechnical University, 29 Polytehnicheskaya Street, St. Petersburg 195251, Russia; b Department of Genetics, St. Petersburg State University, Universitetskaya nab. 7/9, St. Petersburg 199034, Russia; c Chicago Center for Systems Biology, and Department of Ecology and Evolution, The University of Chicago, Chicago, IL 60637, USA; d Department of Applied Mathematics and Statistics, and Center for Developmental Genetics, Stony Brook University, Stony Brook, NY 11794-3600, USA; e Department of Statistics, and Department of Molecular Genetics and Cell Biology, The University of Chicago, Chicago, IL 60637, USA

and

“Lack of *tailless* leads to an increase in expression variability in *Drosophila* embryos” [Dev. Biol. 377 (2013) 305–317]

  Hilde Janssens^a^, Anton Crombach^a^, Karl Richard Wotton^a^, Damjan Cicin-Sain^a^, Svetlana Surkova^b^, Chea Lu Lim^c^, Maria Samsonova^b^, Michael Akam^c^, Johannes Jaeger^a,c,^*

^a^ *EMBL/CRG Research Unit in Systems Biology, CRG—Centre de Regulaciò Genòmica, and Universitat Pompeu Fabra (UPF), Dr. Aiguader 88, 08003 Barcelona, Spain*

^b^ *Department of Computational Biology, Center for Advanced Studies, St. Petersburg State Polytechnical University, 29 Polytehnicheskaya Street, St. Petersburg 195251, Russia*

^c^ *Department of Zoology, Downing Street, Cambridge CB23EJ, UK*

The articles mentioned above were meant to be published back to back as companion papers in the same issue.

  *Corresponding authors.

*E-mail addresses*: samson@spbcas.ru (M. Samsonova), yogi.jaeger@crg.eu, yoginho@gmail.com (J. Jaeger).

