# Supplementary material for: Lack of tailless leads to an increase in expression variability in Drosophila embryos
Source: Dev Biol. 2013 May 1;377(1):305–17. doi: 10.1016/j.ydbio.2013.01.010 (PMC3635121; doi:10.1016/j.ydbio.2013.01.010)
Supplement: Supplementary file 1 — Supplementary data [file mmc1.pdf]

Supplementary Tables

Janssens et al.

Table S1. Number of embryo expression profiles used for data quantification, per gene and per time class. (*) c13 and c14A t1 *tll*$^-$/*tll*$^-$ embryos can only be distinguished from heterozygous or wild-type embryos through lack of Tll expression. Since not all embryos in our data set were stained for Tll, the *tll*^{g} numbers for c13 and t1 below represent a mix of mutant and wild-type embryos. Comparing embryos without Tll staining with wild-type embryos (last 2 columns) shows that there are only very subtle differences in Eve expression in *tll*$^-$/*tll*$^-$ versus wild-type at this stage (see Figure 4).

	**Eve**		**Bcd**		**Cad**		**Gt**		**Hb**		**Hkb**		**Kni**		**Kr**		**Tll**	
**tc**	***tll*^{g}**	**wt**	***tll*^{g}**	**wt**	***tll*^{g}**	**wt**	***tll*^{g}**	**wt**	***tll*^{g}**	**wt**	***tll*^{g}**	**wt**	***tll*^{g}**	**wt**	***tll*^{g}**	**wt**	***tll*^{g}**	**wt**
c13*	63	171	20	98	8	47	1	9	12	49	24	28	29	2	11	21	11	22
t1*	192	182	36	58	27	28	36	21	51	62	83	36	64	15	44	25	18	28
t2	69	112	15	30	13	15	24	20	23	30	15	37	19	14	12	24	13	13
t3	57	162	15	38	7	21	14	23	19	43	11	36	14	16	11	34	14	19
t4	62	149	14	28	7	17	17	24	22	28	17	29	17	12	16	36	13	14
t5	76	180	24	25	13	14	21	27	32	44	15	34	24	22	17	54	16	16
t6	51	170	19	29	3	18	16	21	29	47	11	34	21	20	20	52	15	18
t7	49	157	20	15	6	13	20	23	15	33	10	16	16	20	12	48	7	12
t8	31	144	7	12	3	12	8	25	10	33	3	13	13	17	13	38	6	17

Table S2. Average positions of Eve domain peaks in wild-type and *tll^g* mutant embryos. Average positions (in % along the A–P axis, where 0% is the anterior pole) are shown for the point of maximum intensity of each visible Eve stripe from t3 to t8. Moreover, we show Bonferroni-corrected *p*-values resulting from Student's t-tests for equality of the mean. Shaded boxes highlight p-values indicating significant differences in stripe positions in wild-type compared to mutants (*p<0.005*). See Materials and Methods for details on measurements and statistical tests.

	Stripe1			Stripe2			Stripe3			Stripe4			Stripe5			Stripe6			Stripe7		
tc	wt	*tll^g*	p	wt	*tll^g*	p	wt	*tll^g*	p	wt	*tll^g*	p	wt	*tll^g*	p	wt	*tll^g*	p	wt	*tll^g*	p
3	32.38	31.33	203.552	42.66	42.61	114.943	50.84	51.62	0.242	59.41	59.95	6.685	-	69.25	-	-	81.91	-	83.68	-	-
4	31.94	31.65	158.843	42.40	43.06	7.183	50.87	52.10	0.037	58.18	59.92	9e-05	66.12	69.33	6e-12	72.94	81.75	5e-37	84.08	-	-
5	31.65	30.66	203.964	41.68	41.57	139.019	50.05	50.28	29.702	57.34	58.15	0.030	65.12	67.60	2e-14	72.45	80.12	3e-37	82.75	-	-
6	31.56	30.62	203.659	41.35	40.92	193.095	49.74	49.79	84.960	57.11	57.82	1.156	64.77	67.22	3e-06	72.28	79.81	3e-19	81.84	-	-
7	31.69	31.37	173.173	40.82	41.19	21.202	49.14	49.89	0.549	56.47	57.60	0.004	63.75	66.39	4e-11	71.11	78.87	5e-29	80.26	-	-
8	31.89	30.54	203.556	40.77	40.19	185.095	48.90	48.88	105.582	55.99	56.35	27.182	62.91	65.00	5e-05	70.32	77.60	5e-13	79.32	87.37	5e-11

Table S3. Temporal shifts of Eve domain peaks in wild-type and *tll^g* mutant embryos. This table shows differences between peak positions (in % egg length, EL) at t3 and t8 from Table S2.

	Stripe 1	Stripe 2	Stripe 3	Stripe 4	Stripe 5	Stripe 6	Stripe 7
tc	3-8	3-8	3-8	3-8	4-8	4-8	3-8
wt	0.49	1.89	1.94	3.42	3.21	2.62	4.36
tll^g	0.79	2.42	2.74	3.59	4.34	4.16	-

Table S4. Widths of Eve stripes in wild-type compared to tll^g mutant embryos. Average domain widths (in %EL) are shown for each detectable Eve stripe from t5 to t8. We also show Bonferroni-corrected *p*-values resulting from Student's t-tests for equality of the mean domain size. Shaded boxes highlight those *p*-values indicating significant differences in domain width between wild-type and mutant embryos (*p<0.005*).

	**Stripe 1**			**Stripe 2**			**Stripe 3**			**Stripe 4**			**Stripe 5**			**Stripe 6**			**Stripe 7**		
**tc**	**wt**	tll^g	p	**wt**	tll^g	p	**wt**	tll^g	p	**wt**	tll^g	p	**wt**	tll^g	p	**wt**	tll^g	p	**wt**	tll^g	p
5	5.15	4.89	23.820	5.55	5.38	22.366	4.23	4.05	23.642	3.84	3.90	5.995	4.70	4.55	21.883	5.02	7.01	5e-15	6.94	-	-
6	4.39	4.31	18.672	5.15	4.98	22.164	4.11	3.92	23.273	3.72	4.15	0.002	4.06	4.69	6e-05	4.56	7.37	5e-15	6.45	-	-
7	3.64	3.36	23.966	4.46	3.95	23.998	3.74	3.59	23.275	3.60	3.85	0.152	3.77	4.27	2e-05	4.23	8.24	5e-15	5.63	-	-
8	3.14	2.96	22.589	3.74	3.01	24.000	3.32	3.11	23.136	3.35	3.47	3.694	3.52	3.65	4.265	3.86	8.68	5e-15	5.27	-	-

Table S5. Variability in the positions of Eve stripe peaks in wild-type and tll^g mutant embryos. Standard deviations (in %EL) are shown for the point of maximum intensity of each detectable Eve stripe from t3 to t8. We also show Bonferroni-corrected *p*-values resulting from Levene's tests for equality of variances. Shaded boxes highlight those *p*-values indicating significant differences in variability between wild-type and mutant embryos (*p<0.005*).

	**Stripe 1**			**Stripe 2**			**Stripe 3**			**Stripe 4**			**Stripe 5**			**Stripe 6**			**Stripe 7**		
**tc**	**wt**	tll^g	**p**	**wt**	tll^g	**p**	**wt**	tll^g	**p**	**wt**	tll^g	**p**	**wt**	tll^g	**p**	**wt**	tll^g	**p**	**wt**	tll^g	**p**
3	1.55	2.44	0.012	1.41	1.97	0.085	1.27	1.57	0.328	1.34	1.86	0.200	-	2.21	-	-	2.97	-	1.62	-	-
4	1.48	2.68	0.019	1.33	2.59	0.001	1.12	2.43	2e-05	1.08	2.39	1e-04	1.23	2.59	0.011	1.27	2.61	3e-04	1.67	-	-
5	1.50	2.02	3.529	1.31	1.87	9.202	1.09	1.67	2.753	0.99	1.70	0.007	1.08	2.03	2e-06	1.23	2.83	9e-11	1.68	-	-
6	1.45	1.99	0.147	1.26	1.68	3.188	1.11	1.63	1.588	1.11	1.80	0.131	1.34	2.59	1e-03	1.49	3.51	9e-07	1.82	-	-
7	1.45	1.92	1.538	1.34	1.80	2.044	1.18	1.62	3.737	1.16	1.58	18.591	1.23	1.87	0.052	1.40	2.37	0.085	1.57	-	-
8	1.60	2.28	17.921	1.40	2.22	3.361	1.17	1.88	0.106	1.14	1.64	2.251	1.12	1.80	0.159	1.26	2.96	8e-06	1.34	2.48	0.242

Table S6. Variability in widths of Eve stripes in wt and *tll*^{g} mutant embryos. Standard deviations (in %EL) are shown for the width of each detectable Eve stripe from t5 to t8. We also show Bonferroni-corrected *p*-values resulting from Levene's tests for equality of variances. Shaded boxes highlight those *p*-values indicating significant differences in width variability between wild-type and mutant embryos (*p<0.005*).

	Stripe 1			Stripe 2			Stripe 3			Stripe 4			Stripe 5			Stripe 6			Stripe 7		
tc	wt	*tll*^{g}	p	wt	*tll*^{g}	p	wt	*tll*^{g}	p	wt	*tll*^{g}	p	wt	*tll*^{g}	p	wt	*tll*^{g}		wt	*tll*^{g}	p
5	0.69	0.75	8.671	0.60	0.81	0.144	0.41	0.64	0.002	0.48	0.64	0.344	0.78	0.72	12.950	0.70	1.63	9e-09	0.64	-	-
6	0.50	0.73	0.119	0.46	0.80	1e-05	0.39	0.67	0.045	0.36	0.73	6e-07	0.46	0.86	0.001	0.48	1.59	3e-13	0.73	-	-
7	0.47	0.54	3.900	0.54	0.81	0.807	0.36	0.50	0.572	0.34	0.66	0.016	0.39	0.60	0.095	0.40	1.61	1e-13	0.57	-	-
8	0.48	0.57	1.274	0.58	0.57	9.730	0.38	0.58	0.001	0.49	0.58	0.539	0.48	0.70	0.135	0.48	1.56	4e-07	0.57	-	-

Table S7. Average positions of peaks and boundary positions of gap domains in wild-type and *tll*^{g} mutant embryos. Average positions (in % along the A–P axis, where 0% is the anterior pole) are shown for the position of the anterior boundary, the maximum intensity peak, and the posterior boundary of the central Kr, the posterior Kni, and posterior Gt domain from t3 to t8. See Materials and Methods for details on measurements of boundary and peak positions.

	Central Kr						Posterior Kni						Posterior Gt					
	A boundary		Peak		P boundary		A boundary		Peak		P boundary		A boundary		Peak		P boundary	
tc	wt	*tll*^{g}	wt	*tll*^{g}	wt	*tll*^{g}	wt	*tll*^{g}	wt	*tll*^{g}	wt	*tll*^{g}	wt	*tll*^{g}	wt	*tll*^{g}	wt	*tll*^{g}
3	44.39	46.21	52.15	53.06	60.16	61.11	59.01	61.39	64.80	66.71	70.68	74.04	69.33	73.12	75.00	83.36	82.26	91.70
4	44.22	47.42	52.13	55.05	60.11	62.78	58.82	62.25	64.03	67.32	69.82	74.83	68.67	73.71	74.14	80.96	79.77	89.65
5	43.91	45.34	51.62	52.42	59.56	61.16	58.39	60.70	63.81	66.05	69.38	72.33	68.01	74.17	73.00	80.37	78.48	87.38
6	43.61	45.40	51.15	51.81	59.19	60.80	58.39	59.62	63.57	65.67	68.99	71.66	67.32	72.50	72.22	78.10	77.51	85.12
7	43.22	44.61	50.18	50.57	57.87	57.86	57.85	59.38	63.06	65.08	68.34	71.03	66.82	72.32	71.35	78.45	76.36	84.61
8	43.28	44.85	49.89	49.96	57.16	56.05	57.14	7.33	62.60	63.14	67.65	69.03	66.29	70.03	70.66	77.44	75.48	84.4

Table S8. Temporal shifts of gap domain peaks in wild-type and *tll*^{g} mutant embryos. The values shown in this table represent differences between peak positions (in %EL) at t3 and t8 from Table S7.

	Central Kr		Posterior Kni		Posterior Gt	
tc	wt	*tll*^{g}	wt	*tll*^{g}	wt	*tll*^{g}
3-8	2.26	3.10	2.21	3.57	4.34	5.92

Table S9. Widths of gap domains in wild-type and *tll*^{g} mutant embryos. Average domain widths (in %EL) are shown for the central Kr, the posterior Kni, and the posterior Gt domain from t3 to t8.

	Central Kr		Posterior Kni		Posterior Gt	
tc	wt	*tll*^{g}	wt	*tll*^{g}	wt	*tll*^{g}
3	15.77	14.89	11.67	12.65	12.92	18.58
4	15.89	15.37	11.00	12.58	11.09	15.94
5	15.65	15.82	10.98	11.63	10.47	13.21
6	15.57	15.39	10.61	12.04	10.19	12.62
7	14.64	13.25	10.50	11.65	9.54	12.29
8	13.88	11.20	10.52	11.70	9.19	14.46

Table S10. Variability in the position of gap domain peaks and boundaries in wild-type and *tll*^{g} mutant embryos. Standard deviations (in %EL) are shown for the position of the anterior boundary, the peak of maximum intensity, and the posterior boundary of the central Kr, the posterior Kni, and posterior Gt domain from t3 to t8.

	Central Kr						Posterior Kni						Posterior Gt					
	A boundary		Peak		P boundary		A boundary		Peak		P boundary		A boundary		Peak		P boundary	
tc	wt	*tll*^{g}	wt	*tll*^{g}	wt	*tll*^{g}	wt	*tll*^{g}	wt	*tll*^{g}	wt	*tll*^{g}	wt	*tll*^{g}	wt	*tll*^{g}	wt	*tll*^{g}
3	1.39	1.52	1.30	1.22	1.25	1.35	0.96	2.27	0.97	2.46	1.02	3.67	1.03	2.55	0.99	5.66	1.79	6.15
4	1.24	1.93	1.14	2.12	1.20	2.42	1.05	2.56	0.80	2.86	0.88	2.72	1.16	1.33	1.47	1.62	1.81	2.96
5	1.03	1.65	1.00	2.24	1.05	2.22	1.00	2.36	0.95	3.05	1.18	2.61	0.97	1.01	1.24	1.62	1.48	2.07
6	1.12	1.62	0.93	1.75	1.19	2.30	0.89	2.04	0.80	2.16	1.06	2.69	0.94	2.34	1.09	1.09	1.33	1.47
7	1.20	2.02	1.22	1.80	1.43	2.46	0.89	1.68	1.05	1.57	1.04	1.66	0.94	2.43	1.15	2.82	1.07	3.50
8	1.09	1.91	0.95	2.01	1.42	1.20	0.78	1.35	0.92	1.45	1.01	2.03	1.08	2.46	1.16	1.73	1.22	2.98

Table S11. Variability in widths of gap domains in wild-type and *tll^g* mutant embryos. Standard deviations (in %EL) are shown for the width of the central Kr, the posterior Kni, and posterior Gt domain from t3 to t8.

	Central Kr		Posterior Kni		Posterior Gt	
**tc**	**wt**	***tll^g***	**wt**	***tll^g***	**wt**	***tll^g***
3	1.10	2.25	1.08	2.99	2.07	4.66
4	1.04	1.95	0.79	1.77	1.02	2.82
5	0.91	1.93	0.78	2.19	1.16	1.93
6	1.15	2.44	0.80	2.02	0.83	1.97
7	1.42	1.03	0.51	0.63	0.48	2.01
8	1.29	1.05	0.83	1.64	0.54	4.28
